# What factors impact pedestrian and cyclist fatalities? A state level analysis

**DOI:** 10.1186/s40621-021-00315-z

**Published:** 2021-09-13

**Authors:** Zoabe Hafeez, Malvi Mehta

**Affiliations:** 1 McGovern Medical School at UTHealth, Department of Pediatrics, 1133 John Freeman Blvd, JJL 210C, Houston, TX 77030 USA; 2 McGovern Medical School at UTHealth, 6431 Fannin St, Houston, TX 77030 USA

**Keywords:** Pedestrian safety, Bicyclist safety, Transportation infrastructure, Motor vehicle crashes, Pedestrian mortality, Bicyclist mortality, Vulnerable road users

## Abstract

**Background:**

Pedestrian and bicyclist injuries and fatalities have increased since 2010 after a long downward trend. Trucks and SUVs, collectively called light trucks, have also increased in sales and size, which may affect pedestrians and bicyclists. Additionally, pedestrian and cyclist commuters vary by state and it has been speculated that an increase in such commuters may affect fatalities. Studying vulnerable road users can bestow clues on best practices for infrastructure and public health.

**Methods:**

State level pedestrian and cyclist fatality data was obtained from the National Highway Transportation Safety Administration for 2018. Light truck registration by state was obtained from the Office of Highway Policy Information for 2018. Commuters who walk or bike to work were obtained from the American Community Survey from 2009 to 2011, from the latest Centers for Disease Control report. We performed multiple linear regression, accounting for total motor vehicle lane miles per 100 people, also obtained from the Office of Highway Policy Information for 2018. Multiple regression analysis was performed to assess predictors for pedestrian and cyclist fatalities with the predictors variables of light truck registration, lane miles per 100 people, and proportion of commuters who are vulnerable road users. Secondary analysis included simple linear regression of the predictor variables against each other.

**Results:**

The multiple regression model, including proportion of light truck registration, lane miles per 100 people, and proportion of commuters who are vulnerable road users, accounted for 18% of the variability in the outcome variable (*p* = 0.03). An increased number of vulnerable road users were negatively associated with pedestrian and bicyclist fatality. Additionally, there appeared to be an association between motor vehicle lane miles per 100 people and proportion of light truck registrations that was also significant (*p* < 0.01).

**Conclusion:**

The variables affecting vulnerable road user deaths are important to understand given their increased risk exposure on the road. This state level study identifies a potential protective variable with increased vulnerable road users being associated with a decrease in pedestrian and bicyclist death rates. Additionally, light truck proportions do not appear to have a significant effect on death rates.

## Background

Pedestrian and bicyclist injuries have been rising in the United States despite an increasing public focus on infrastructure for pedestrians and bicyclists, collectively termed ‘vulnerable road users’ in this article. Pedestrian fatalities have increased by more than 50% in the last ten years alone, with light trucks causing 81% of pedestrian deaths in 2018 and 2019 (Governors Highway Safety Association 2020). Furthermore, injuries caused by motor vehicle injuries are a leading cause of death in children and adolescents (Cunningham et al. 2018). Additionally, the proportion of motor vehicles that are defined as light trucks, which include trucks and sport utility vehicles (SUVs), are increasing (Bureau of Transportation Statistics 2020). Some studies have shown an association between these two phenomena, including an American cross sectional study from 2002 (Paulozzi 2005), a retrospective analysis focused on New York City in the 1990s (DiMaggio et al. 2006), and an Australian study summarizing low speed fatalities on young children in the late 1990s (Neeman et al. 2002). The increased morbidity and mortality that light trucks impose on vulnerable road users are likely due to light trucks’ large size, taller body, stiffer front structure, and decreased visibility for their drivers (Karaca-Mandic and Lee 2014).

Americans walk and bike less than many of their first world counterparts (Althoff et al. 2017), however this varies by state (Centers for Disease Control and Prevention 2014). There have been local observations of a ‘safety in numbers’ paradox where the more bicyclists or pedestrians in a locality, the decreased proportion of pedestrian or bicyclist fatalities. This has been shown in California in 2000, Denmark in the 1990s, and in the Netherlands in the 1980s and 1990s through a review article in 2004 (Jacobsen 2015). However, this has been more difficult to replicate in all situations, suggesting infrastructure may also play a role (Marshall and Ferenchak 2019).

The United States is unusually dangerous for road cyclists and pedestrians compared to other first world countries (Cunningham et al. 2018). As such the factors affecting vulnerable road user safety specifically still require further study in the face of worsening pedestrian deaths. This study evaluated the 50 states of the United States and whether there is a relationship between the vulnerable road user fatality, proportion of light trucks, lane miles per 100 people, and proportion of vulnerable road users in each state. To our knowledge, this study is the first state level study of the United States to determine contributing or protective factors associated with pedestrian and cyclist fatality.

## Methods

The primary outcome of interest was pedestrian and bicyclist fatality rate, expressed as pedestrian and bicyclist fatalities per 100,000 people for each state in 2018. The predictor variables were motor vehicle lane miles per 100 people, proportion of motor vehicles that were light trucks, and proportion of the states’ residents who reported walking or biking to work. The aim was to assess whether truck size or number of vulnerable road users were associated with an increase or decrease in pedestrian and bicyclist fatality rate. The variable motor vehicle lane miles per 100 people was measured to account for differences in infrastructure subsidized car dependence in each state. This was used instead of vehicle miles travelled as the former serves as a more accurate indicator of infrastructure allocation, without introducing biases such as economic strength and gas prices (Puentes and Tomer 2008). The light trucks variable was expressed as the proportion of 2018 registrations that were light trucks, to approximate light truck prevalence in the respective state. The commuter variable was expressed as the proportion of state residents who reported walking or biking to work, to approximate vulnerable road user prevalence in the respective state.

Each state’s lane miles in 2018 were obtained by the Federal Highway Administration’s (FHA) Office of Highway Policy Information (Federal Highway Administration Office of Highway Policy Information 2019). The population of each state in 2018 was obtained from the United States Census Bureau (United States Census Bureau n.d.). Light trucks, defined as trucks weighing up to 8500 pounds and a payload capacity of up to 4000 pounds, include typical non-commercial trucks and SUVs. Light truck registrations were obtained as a proportion of total motor vehicle registration in 2018 and were also obtained by the FHA’s Office of Highway Policy Information (Federal Highway Administration Office of Highway Policy Information 2020). Pedestrian and bicyclist fatality rates for 2018 were obtained by the National Highway Traffic and Safety Administration (National Highway Traffic Safety Association 2020). Finally, the percent of the population that walks or bikes to work by state was obtained from the Centers for Disease Control’s 2014 State Indicator Report on Physical Activity. This paper reported information aggregated from the American Community Survey from 2009 to 2011 asking ‘How did this person usually get to work last week?”. Respondents who reported “Bicycle” or “Walked” were classified as a bicyclist or pedestrian commuter (Centers for Disease Control and Prevention 2014).

Multiple regression with an outcome measure of pedestrian and bicyclist fatalities per 100,000 people was performed with all 50 states and Washington D.C. as samples. Washington D.C., the only sample that is a single city and does not hold statehood, had an unusually high influence on the regression model when investigated with a leverage versus squared residual plot and on a comparison of Cook’s distance of all samples. It was elected to be excluded from the dataset as a result. Normality of the residuals was assessed using the Shapiro-Wilk test for normal data as well as the Skewness and Kurtosis test for normality as developed by D’Agostino, Belander, and D’Agostino (D’Agostino et al. 1990) with the adjustment made by Royston (Royston 1992). These revealed no evidence of kurtosis, but a right skew that was corrected by transforming the outcome measure to log scale. While this prevented us from meaningfully interpreting significant coefficients in the regression mode, it was necessary to ensure normality. The Breusch-Pagan test for heteroskedasticity was reassuring as were tests for linearity. Simple linear regression was performed with all predictor variables against the outcome variable to assess for individual R^2^. The data were analyzed using STATA/IC version 16.1 (StataCorp, College Station, TX). Statistical significance was defined as *p* < 0.05.

## Results

**Fig. 1:**
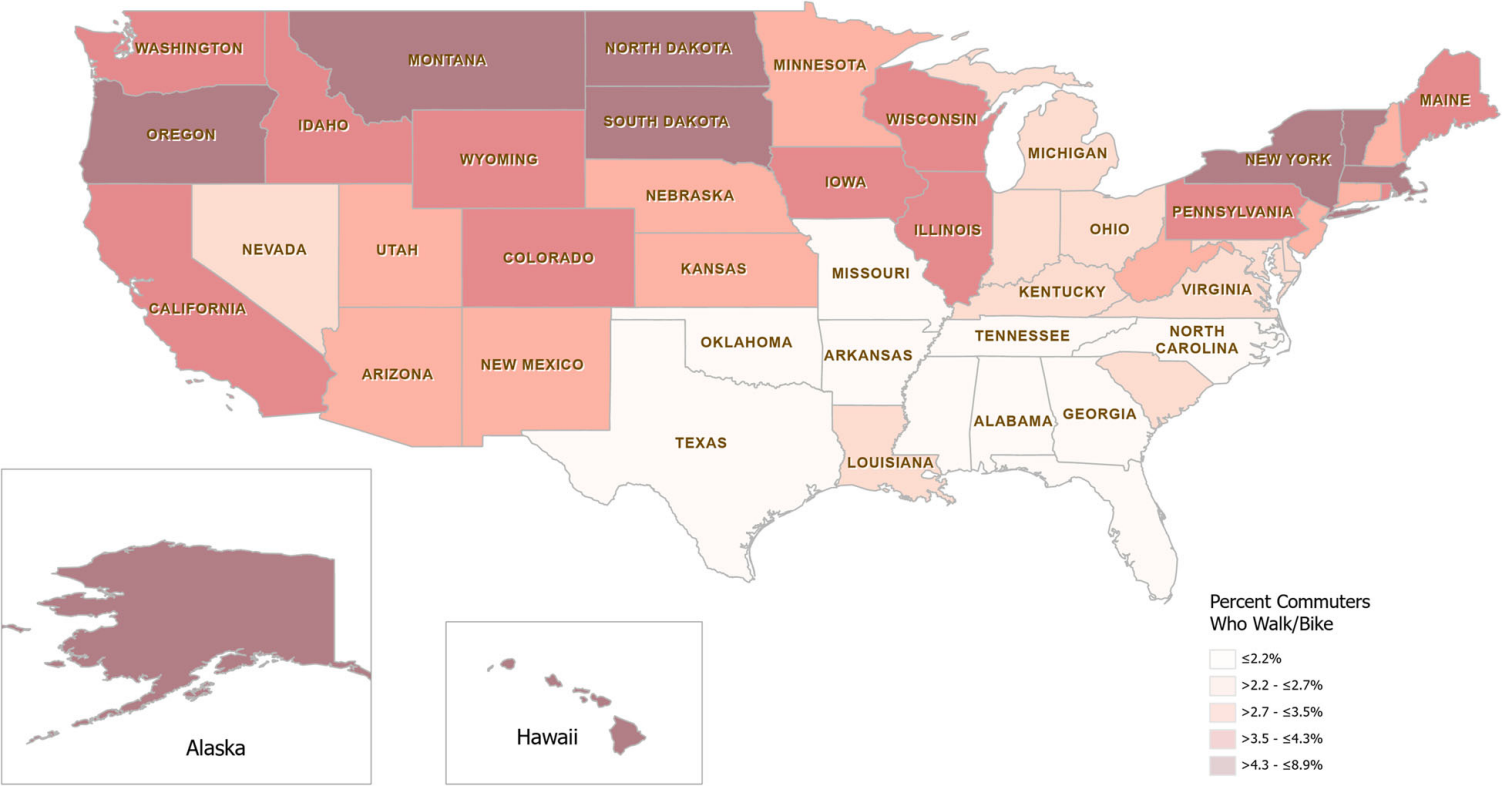
Heat map of percent commuters who walk or bike to work for each state

The percent of commuters that identified as vulnerable road users had a mean of 3.5% for all 50 observations and showed variation between the states, as shown in the heat map in Fig. 1. This ranged from lows of 1.4% in Alabama and 1.5% in Tennessee to highs of 8.9% in Alaska and 6.9% in New York. There is a notable clustering of states with a low proportion of vulnerable road users in the Southern region of the United States.

**Fig. 2:**
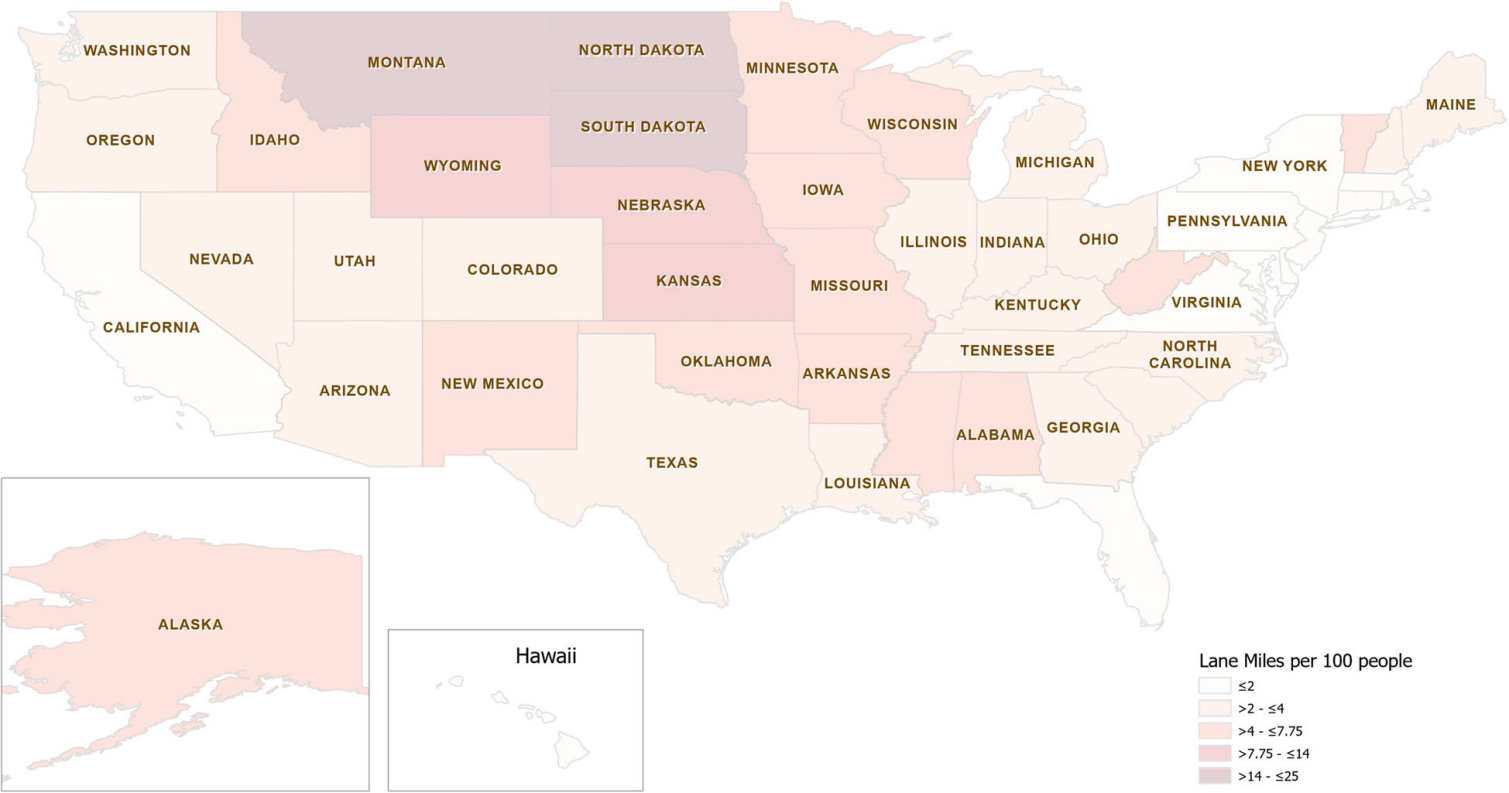
Heat map of lane miles/per 100 people for each state

Lane miles per 100 people by state also displayed variation as shown in the heat map in Fig. 2. The mean was 4.59 miles per 100 people. This ranged from 0.69 miles in Hawaii and 0.96 miles in New Jersey to high values of 19.08 miles and 23.56 miles in South Dakota and North Dakota respectively.

**Fig. 3:**
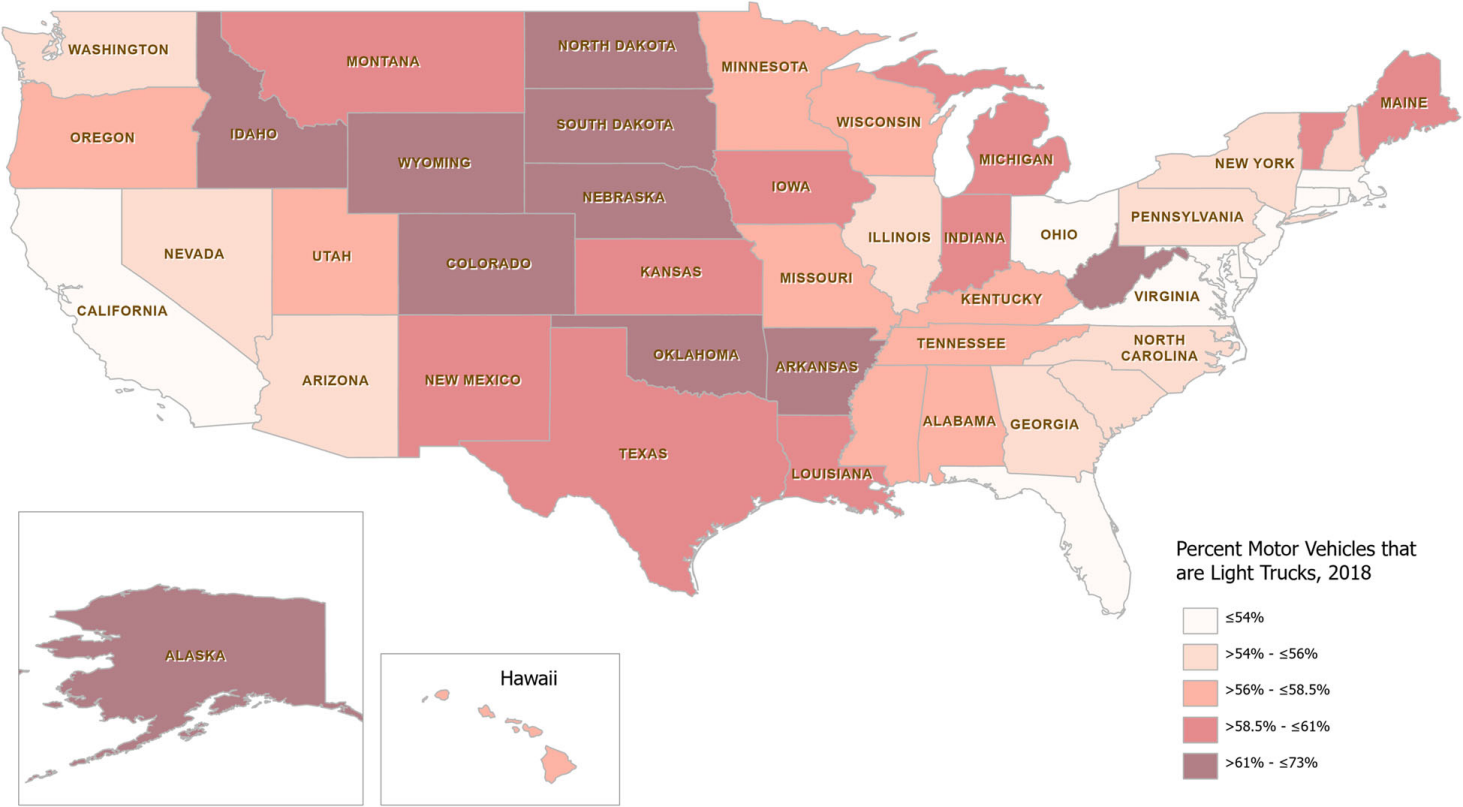
Heat Map of percent of total motor vehicles registered as light trucks, 2018. Divided into Quintiles

Figure 3 shows the quintiles of the proportion of total motor vehicles that were registered as light trucks in 2018 for each state. This was used as a proxy for the prevalence of light trucks on the road, including passenger trucks and SUVs. The mean light truck percentage was 58% with California and Rhode Island on the low end at 48 and 49% respectively. The highest truck proportions were found in Alaska and Wyoming at 72% each. Truck heavy states were concentrated in the Great Plains region of the country.

This appears visually similar to the lane miles heat map, however tests for multicollinearity were unremarkable (Variance Inflation Factor was < 2). Notably, on a simple linear regression analysis of truck registration proportion and lane miles per 100 people, 38% of the variance between states in truck registration proportion could be explained by the lane miles metric. A summary of the four variables are available in Table 1 below.

**Table 1:** Description of variables

Variable	Mean	Standard Deviation	Min Value (State)	Max Value (State)
Pedestrian and Bicyclist Fatality per 100,000 people^a^	1.95	0.94	0.67 (Maine)	4.49 (New Mexico)
Percent of Commuters - Walk or Bike to Work	3.54	1.55	1.4 (Alabama)	8.9 (Alaska)
Lane Miles Per 100 people	4.59	4.49	0.69 (Hawaii)	23.6 (North Dakota)
Proportion of Motor Vehicles Registered as Trucks	0.58	0.05	0.48 (California)	0.72 (Alaska)

^a^ Pedestrian and Bicyclist Fatality rate was logarithmically transformed for the regression model

Multiple regression analysis found a significant association between these variables (p = 0.028) with 18% of the variability between states in vulnerable road user fatality incidence in 2018 determined by these three variables. In particular, the number of vulnerable road users appeared to have a protective effect on vulnerable road user mortality rates, indicated by a negative coefficient. Lane miles per 100 people also had a negative coefficient, but was not significant within the multiple regression model. However, it was a contributor to the model when there was a corresponding and significant increase in R^2^ compared to when it was not included in the model.

In the multiple regression model, only the proportion of commuters that walk or bike to work was significant with *p* < 0.05 (Table 2), however the multiple regression model explained a greater proportion of the variance (R^2^ = 18%, *p* = 0.03) than the simple linear regression model with only the outcome variable and the proportion of commuters that walk or bike (R^2^ = 12%, *p* = 0.01). Additionally, the relationship between the outcome variable and lane miles per 100 people for each state was significant in a simple linear regression model (*p* = 0.04), with a negative coefficient indicating that more lane miles per person was associated with a decreased mortality rate. Finally, the proportion of truck registrations was not significant when associated with the outcome variable neither within the multiple regression model nor in the simple linear regression model.

**Table 2:** Multiple linear regression analysis of all variables with outcome variable of the logarithmically transformed pedestrian and bicyclist fatality rate. R^2^ = 0.178, F = 3.32, *p* = 0.028

Variable	Coefficient	Standard Error	t	*p* value	95% Confidence Interval	
Proportion of Truck Registrations	0.345	1.649	0.21	0.835	−2.97	3.66
Lane Miles per 100 People	−0.028	0.0180	−1.54	0.129	−0.064	0.008
Proportion of Commuters Walk or Bike to Work	−0.095	0.043	−2.23	0.030	−0.181	−0.009

## Discussion

The primary hypothesis of this study was to evaluate if there was a relationship between fatality rates of vulnerable road users and proportion of light trucks on the road, lane miles per 100 people, and proportion of vulnerable road users at a state level. We found that there is a relationship that accounts for 18% of the variability between states. The most significant variable affecting fatality rates was the proportion of vulnerable road users. This gives further credence to the growing body of evidence that suggests that there is a ‘safety in numbers effect’ on roadways (Jacobsen 2015; Marshall and Ferenchak 2019; Murphy et al. 2017). That is, the more bicyclists and pedestrians in an environment, the safer it is for bicyclists and pedestrians. This could be due to increased awareness of motor vehicle users to vulnerable road users in those environments (Jacobsen 2015). However, other factors may also be involved, such as better bicycling and pedestrian infrastructure development leading to both more vulnerable road users as well as increased safety. Perceived safety has been cited as a common determinant of whether commuters choose to bicycle to work (Wachtel and Lewiston 1994), so a safer appearing road may contribute to fewer deaths as well as more pedestrians and bicyclists independently. Additionally, the presence of vulnerable road users may also slow motor vehicle speeds, a known contributor to pedestrian and bicyclist fatalities (Anderson et al. 1997).

Lane miles as a proportion of the population also contributed to the multiple regression model and was significantly associated with fatality rates of vulnerable road users. One possible explanation is the concentration of states with high lane miles per person are in sparsely populated areas of the country. States like Montana, North Dakota, South Dakota, Wyoming, and Nebraska may simultaneously have low mortality rates and high lane miles per person simply due to their small population and extremely low density.

The proportion of light truck registrations did not seem to be an explanatory variable for the variability of vulnerable road user deaths at a state level in 2018. However, light truck registrations were significantly associated with lane miles per person. This may be explained by the phenomenon of the built environment contributing to user preference.

This study is the first state level analysis looking at the association between the mortality rate of vulnerable road users and the factors that have historically been implicated in affecting it. The aforementioned ‘power in numbers’ phenomenon is supported in this research, however the proportion of large vehicles adversely affecting vulnerable road users, a phenomenon previously reported in the literature, does not seem to be a significant factor in this study.

This study had limitations. First, this is a state level study and variables affecting vulnerable road users such as transportation infrastructure, traveler habits, and public safety initiatives can vary greatly even on the local level. While this study serves as a state level ecological study of our nation, more studies on a local level can add much to local policy and public health initiatives through a more granular approach accounting for local variation in the interplay of these, and other, variables. Second, light truck registration is meant to serve as the proportion of trucks and SUVs on the road in each state. It is possible that the actual distribution of motor vehicles is different in some states if purchase trends change over time or if there is a sizable proportion of unregistered motor vehicles. Finally, despite our best efforts, we were not able to obtain sizable state level commuter data later than 2009 to 2011, compared to the other variables which were obtained in 2018. The time that the commuter data was obtained is an obvious limitation on this study and should be accounted for in its interpretation. However, we believe that the proportions included in this study are representative of a more robust sample size, as they included multiple years of data, decreasing the likelihood of variability from a smaller sample size. Additionally, it should be noted that this study used commuting share as a marker for vulnerable road users. This only represents a fraction of all vulnerable road users, as many bike and walk recreationally, though it is not systematically recorded as commuting bicyclists are. For example, one study of cities in the Western United States indicated that the proportion of bicyclists who usually bike to work range from 0 to 32% depending on the city (Xing et al. 2010).

## Conclusions

Vulnerable road users appear to be growing in their risk exposure on the road (Governors Highway Safety Association 2020) and there is a public health imperative to analyze the variables affecting this increasing susceptibility to morbidity and mortality. This study demonstrated a state level association between increased pedestrians and bicyclists on the road and decreased vulnerable road user mortality rate, supporting a theory of ‘power in numbers’. The proportion of light trucks on the road does not seem to be significantly associated with vulnerable road user mortality rate in this study, which contributes a null finding to the literature. Notably, there was an association between lane miles per person and proportion of light trucks, indicating that consumers may buy their vehicles to match their transportation infrastructure. More research, especially on a census tract or block group level, can help further elucidate the association between these four variables to aid public health experts and their communities.

## Data Availability

The datasets used and/or analyzed during the current study are available from the corresponding author on reasonable request.

## Abbreviations

SUV: Sport Utility Vehicle
FHA: Federal Highway Administration
